# Hakai reduces cell-substratum adhesion and increases epithelial cell invasion

**DOI:** 10.1186/1471-2407-11-474

**Published:** 2011-11-03

**Authors:** Teresa Rodríguez-Rigueiro, Manuel Valladares-Ayerbes, Mar Haz-Conde, Luis A Aparicio, Angélica Figueroa

**Affiliations:** 1 Translational Cancer Research Group, Instituto de Investigación Biomédica A Coruña (INIBIC), Complejo Hospitalario Universitario A Coruña (CHUAC), SERGAS, A Coruña, Spain; 2 Servizo Oncología Médica, Complejo Hospitalario Universitario A Coruña (CHUAC), SERGAS, A Coruña, Spain

## Abstract

**Background:**

The dynamic regulation of cell-cell adhesions is crucial for developmental processes, including tissue formation, differentiation and motility. Adherens junctions are important components of the junctional complex between cells and are necessary for maintaining cell homeostasis and normal tissue architecture. E-cadherin is the prototype and best-characterized protein member of adherens junctions in mammalian epithelial cells. Regarded as a tumour suppressor, E-cadherin loss is associated with poor prognosis in carcinoma. The E3 ubiquitin-ligase Hakai was the first reported posttranslational regulator of the E-cadherin complex. Hakai specifically targetted E-cadherin for internalization and degradation and thereby lowered epithelial cell-cell contact. Hakai was also implicated in controlling proliferation, and promoted cancer-related gene expression by increasing the binding of RNA-binding protein PSF to RNAs encoding oncogenic proteins. We sought to investigate the possible implication of Hakai in cell-substratum adhesions and invasion in epithelial cells.

**Methods:**

Parental MDCK cells and MDCK cells stably overexpressing Hakai were used to analyse cell-substratum adhesion and invasion capabilities. Western blot and immunofluoresecence analyses were performed to assess the roles of Paxillin, FAK and Vinculin in cell-substratum adhesion. The role of the proteasome in controlling cell-substratum adhesion was studied using two proteasome inhibitors, lactacystin and MG132. To study the molecular mechanisms controlling Paxillin expression, MDCK cells expressing E-cadherin shRNA in a tetracycline-inducible manner was employed.

**Results:**

Here, we present evidence that implicate Hakai in reducing cell-substratum adhesion and increasing epithelial cell invasion, two hallmark features of cancer progression and metastasis. Paxillin, an important protein component of the cell-matrix adhesion, was completely absent from focal adhesions and focal contacts in Hakai-overexpressing MDCK cells. The expression of Paxillin was found to be regulated by a proteasome-independent mechanism, possibly due to the decreased abundance of E-cadherin.

**Conclusions:**

Taken together, these results suggest that Hakai may be involved in two hallmark aspects of tumour progression, the lowering cell-substratum adhesion and the enhancement of cell invasion.

## Background

The most frequent types of tumours are carcinomas, which develop through the transformation of epithelial cells. Epithelial cells are polarized and are often connected with each other via cell-cell adhesions to form structured cells sheets. The formation of these tight and compact cell-cell adhesions restricts cell movement within the epithelium. Epithelial cells are also attached to an extracellular matrix substratum which is essential for their differentiation and polarization [1]. During transformation, epithelial cells start to proliferate, acquire the ability to migrate, and lose both the intercellular adhesion, mediated by cadherins at adherens junctions, and the interactions with the extracellular matrix. All of these characteristics facilitate the invasion and metastasis of epithelial cells [2].

Hakai was firstly described as a RING finger-type E3 ubiquitin-ligase for the E-cadherin complex [3]. E-cadherin is the prototypical and best characterized member of the cadherins at adherens junctions and its loss is in carcinoma associated with poor prognosis [4]. Hakai binds to the cytoplasmic domain of E-cadherin and mediates its ubiquitination, endocytosis and degradation [3,5]. In consequence, it leads to the disruption of epithelial cell-cell adhesions, inducing epithelial-mesenchymal transition (EMT), a key event in epithelial transformation [6,7]. Apart from this functional role, we recently reported that Hakai is not only implicated in lowering cell-cell contacts, but can also promote proliferation in an E-cadherin-independent manner. Moreover, Hakai overexpression induces anchorage-independent cell growth, produces protrusions that are dynamically extended and retracted, and increases the ability of the RNA-binding protein PSF to bind to mRNAs that encode cancer-related proteins [8,9].

Given the role of Hakai in tumorigenesis, we are interested to examine the possible implication of Hakai in the regulation of adhesions to the extracellular matrix (ECM) and invasion in epithelial cells, two hallmark processes in cancer and metastasis [10,11]. To investigate this possibility, we studied Hakai's influence on cell attachment to the substrate and invasion capacity of Madin-Darby canine kidney (MDCK) cells. We report that in this system, Hakai overexpression leads to a reduction in cell adhesion to the substrate with impact on the key focal adhesion-associated protein Paxillin, and increases cell invasion. Our data suggest that Hakai may have an important functional role during oncogenesis through its implication on cell adhesion to the substrate and cell invasion.

## Methods

### Antibodies and materials

The rabbit polyclonal anti-Hakai antibody (Hakai-2498) was kindly provided by Yasuyuki Fujita [8]. Anti-FAK antibody was from Upstate (Charlottesville, VA), anti-Paxillin antibody was from Transduction Laboratories, anti-Vinculin was from Chemicon International (Hampshire, UK), antibody to the extracellular portion of E-cadherin (ECCD-2) was from Zymed (South San Francisco, CA), anti-α-Tubulin antibody was from Immunologicals Direct (Oxford Biotechnology Ltd., UK), HRP-rabbit and mouse polyclonal antibodies was from GE Healthcare (UK) and Alexa-Fluor 488 mouse antibody and Alexa-mouse 568 were from Molecular Probes (Paisley, UK). All primary antibodies were used at dilutions of 1:1000 for Western blotting and 1:100 for immunofluorescence, except for anti-FAK antibody that was used at a dilution of 1:5000 for Western blotting and 1:500 for immunofluorescence. Secondary antibodies were used at dilutions 1:10000 for HRP-rabbit, 1:5000 for HRP-mouse antibody, and 1:200 for Alexa-Fluor antibodies. pcDNA-HA-Hakai was kindly provided by Walter Birchmeier [3]. MG132 and lactacystin were obtained from Calbiochem (Darmstadt, Germany).

### Cell culture

MDCK cells were cultured in Dulbecco's modified Eagle's medium (DMEM) containing penicillin/streptomycin and 10% FCS at 37°C and ambient air supplemented with 5% CO_2_. MDCK cells stably expressing Hakai were kindly provided by Yasuyuki Fujita [8]. MDCK cells were transfected with pcDNA-HA-Hakai using Lipofectamine-Plus™ reagent (Invitrogen) and were selected in a medium containing 800 μg ml^-1 ^of G418 (Calbiochem). More than ten stable clones were obtained from two independent transfections. All clones, including 4, 6, 7 and 8 clones, represented comparable phenotypes and the results shown were obtained using clone 4, unless otherwise indicated. MDCK cells stably expressing E-cadherin shRNA in a tetracycline-inducible manner were kindly provided by Yasuyuki Fujita [12].

### Adhesion assay

For the adhesion assay, 1 × 10^5 ^cells were plated in a 6-well plate using human fibronectin-coated coverslips (BD Bioscience, Belgium). After 24 hours, the cells were vigorously washed five times with phosphate-buffered saline (PBS), and the remaining cells attached to the plate were analyzed by phase contrast images using a Leica DMIRB microscope with a Hamamatsu C4742-95 Orca camera.

### Western blotting

Cells cultured in 6-well plate dishes were lysed for 30 min in 0.3 ml of 1% Triton X-100 lysis buffer (20 mM Tris-HCL [pH 7.5], 150 mM NaCl, and 1% Triton X-100) containing 5 μg ml^-1 ^lupeptin, 50 mM phenylmethylsulfonyl fluoride, and 7.2 trypsin inhibitor units for aprotinin. After centrifugation at 14, 000 × g for 10 min, thirty micrograms of the supernatants were loaded in 10% polyacrilamide SDS-PAGE. Western blotting was performed as previously described [13].

### Immunofluorescence

For immunofluorescence, human fibronectin-coated coverslips (BD Bioscience, Belgium) were used. Cells on coverslips were washed twice in PBS and incubated in 3% parafolmaldehyde-PBS for 15 min. After washing twice in PBS, cells were permeabilized in 0.5% Triton X-100-PBS for 15 min, followed by blocking in DMEM containing 10% fetal bovine serum for 1 h. They were further incubated in primary antibodies for 1 h, washed in PBS, incubated in Alexa-Fluor 488 or Alexa-Fluor 568-conjugated secondary antibody solution for 1 h, and washed four times in PBS. The coverslip was the mounted onto Mowiol on a glass slide. Immunofluorescent images were analyzed by epifluorescence microscopy. To obtain epifluorescence images, we used a Zeiss Axioskop 1 with a Roper Scientific Coolsnap camera.

### Invasion assay

For invasion assays, 5 × 10^4 ^cells were plated in DMEM/1% FCS in a cell invasion chamber (Cell invasion assay kit, Chemicon International) in a 24-well plate, which contains an 8-μm pore size polycarbonate membrane covered with a thin layer of collagen matrix. Invasive cells migrate through a membrane according to the gradient of FCS to the lower chamber that contains DMEM/10% FCS. The invaded cells were stained with crystal violet, and the absorbance of stained cells was measured in a 565-nm wavelength.

### Statistical analysis

Student *t *test assuming unequal variance was performed for statistical analysis.

## Results and Discussion

### Hakai overexpression reduces cell-substratum adhesion

**Figure 1:**
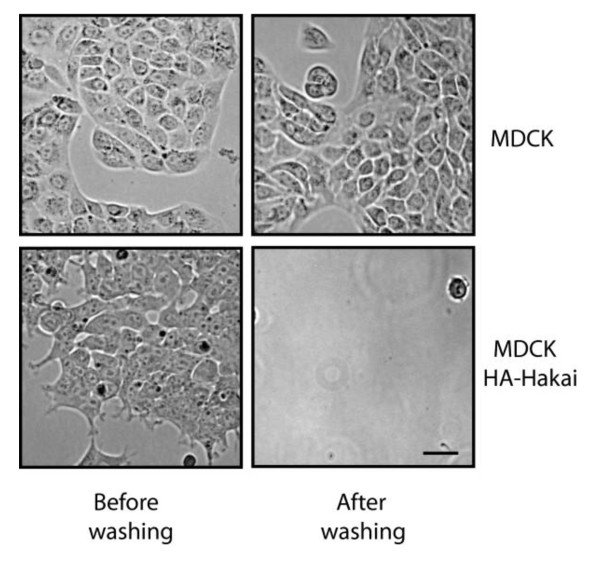
**Effect of Hakai overexpression on the adhesion to the substrate in parental and Hakai-overexpressing MDCK cells**. Cells were plated on human fibronectin-coated coverslips and after 24 h of plating they were vigorously washed 5 times with PBS, and examined by phase contrast. Scale bars, 10 μm.

In a recent study, we reported that MDCK cell lines overexpressing Hakai (4-6-fold higher than endogenous Hakai) exhibited reduced cell-cell contacts and higher cell proliferation than non-transfected parental MDCK cells [8]. Here, we examined the effect of Hakai overexpression on cell-substratum adhesions. When we incubated several clones of Hakai-overexpressing cells, we realized that they had reduced adhesions to the substratum. Parental MDCK cells attached tightly to the substratum and washes with PSB did not detach the cells from the plate (Figure 1, upper panel). In these cells, trypsin was required to detach them from the plate. In contrast, Hakai-overexpressing MDCK cells were easily detached from the plate by washing with PBS (Figure 1, lower panel). This observation suggests that overexpression of Hakai leads to a reduction of cell-substratum adhesions.

**Figure 2:**
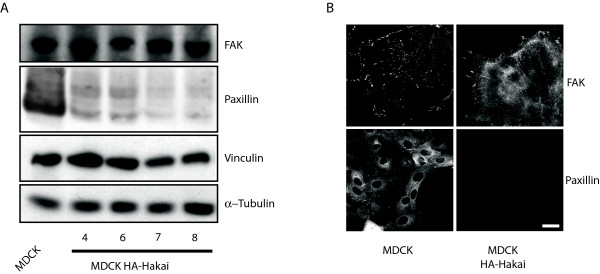
**Effect of overexpression of Hakai on the levels and localization of FAK, Paxillin and Vinculin**. (A) Expression of FAK, Paxillin and Vinculin in parental and in several Hakai-overexpressing MDCK checked clones (described in methods). Lysates prepared from the indicated cells were examined by Western blot analysis to detect the proteins shown. (B) Effect of overexpression of Hakai on the localization of FAK and Paxillin. Parental and Hakai-overexpressing MDCK cells were examined by immunofluorescence with anti-FAK and anti-Paxillin antibodies. Scale bars, 10 μm.

Then, we examined key protein components of cell adhesion, such as FAK, Paxillin and Vinculin, which localize to focal adhesions and focal contacts, mediate adhesion to the substratum, and participate in initiating signal transduction pathways [14]. By Western blotting, we found that Paxillin expression was strongly reduced in Hakai-overexpressing cells, whereas the levels of FAK and Vinculin were not affected by Hakai expression (Figure 2A). Further analysis of the adhesions by immunofluorescence revealed that FAK protein localized at focal adhesions and focal contacts in parental MDCK cells, while in Hakai-overexpressing cells, FAK was diffusely distributed in the cytoplasm, enriched at the sites of focal contacts, and absent from focal adhesions (Figure 2B). As expected, in Hakai-overexpressing cells, Paxillin was undetectable (Figure 2B). These data suggest that cell-substratum adhesions and the formation of focal adhesions are highly dependent on overexpression of Hakai, probably due to the specific downregulation of Paxillin. Although Paxillin primarily functions as a molecular adapter or scaffold protein that provides multiple docking sites at the plasma membrane, it is also important for the transduction of external signals to elicit changes in cell motility and modulate gene expression by the various MAP kinase cascades. Paxillin binds to many proteins that are involved in effecting changes in the organization of the actin cytoskeleton, and are thus necessary for cell motility associated with tumor metastasis [15]. Therefore, the complete absence of Paxillin at focal adhesions may be one of the mechanisms that explain the loss of cell adhesion to the substrate.

### Paxillin down-regulation is controlled by a proteasome-independent mechanism and results from the decreased expression of E-cadherin

**Figure 3:**
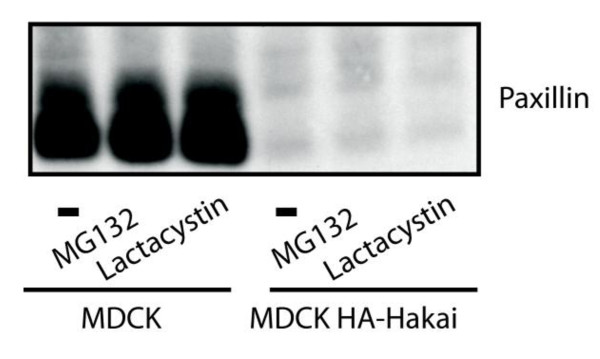
**Effect of proteasome inhibitors on Paxillin expression**. Parental or Hakai-overexpressing MDCK cells were incubated for 6 h in the absence or presence of proteasome inhibitors (20 μM MG132 or 5 μM lactacystin), and cell lysates were examined by Western blotting to detect Paxillin.

**Figure 4:**
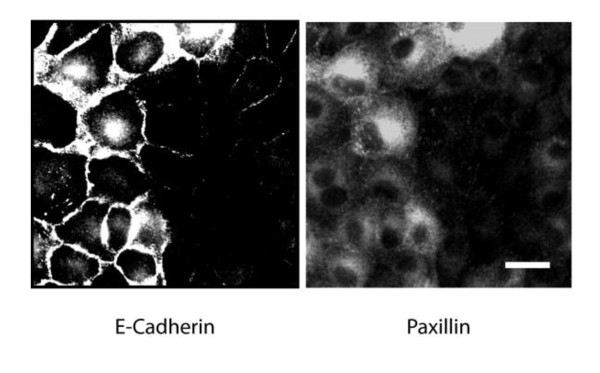
**Effect of E-cadherin knockdown on Paxillin expression**. MDCK pTR cells stably expressing E-cadherin shRNA were incubated for 3 days in the presence of tetracycline, and were analyzed by immunofluorescence to detect E-cadherin and Paxillin. The expression of E-cadherin shRNA is induced only in 30-40% of total cells in this cell line, and the knockdown of E-cadherin is observed in the right half of the presented image. Data are representative of three independent experiments. Scale bar, 20 μm.

To investigate whether the reduced Paxillin levels found in Hakai-overexpressing cells was the result of increased degradation of Paxillin protein, we analyzed the effect of proteasome inhibitors MG132 and lactacystin in Hakai-overexpressing and parental MDCK cells. None of these inhibitors affected Paxillin abundance in Hakai-overexpressing cells (Figure 3), indicating that the expression of Paxillin is controlled in a proteasome-independent manner. We therefore ask whether the loss of E-cadherin was sufficient to reduce Paxillin expression levels. Using MDCK cells in which E-cadhering was knocked down by shRNA in a tetracycline-regulated manner, Paxilllin expression was strongly reduced where E-cadherin was silenced (Figure 4). These findings suggest that the loss of Paxillin in Hakai-overexpressing cells may, at least partially, result from the decreased expression level of E-cadherin.

However, several lines of evidence suggest that Hakai can also affect cellular phenotypes in an E-cadherin-independent manner. First, MDCK cells expressing E-cadherin shRNA do not extend spiky protrusions that are seen in Hakai-overexpressing cells [12]. Second, in Hakai-overexpressing MDCK cells, Hakai is localized at the end of the protrusion where FAK is also enriched [8], suggesting that Hakai may be involved in regulating the dynamic extension and retraction of these structures and in influencing cell motility and cell attachment to the ECM. Finally, Hakai is also located in the nucleus, where it can interact with nuclear proteins such as PSF and modulate cancer-related gene expression [8] and it may act as a correpresor of the estrogen receptor in breast cancer cells [16]. These findings suggest that Hakai can have ubiquitin-independent functions that may influence the cell phenotype, in addition to its influence on known substrates (like E-cadherin) and as-yet unreported substrates in the nucleus or the cytoplasm, as previously reported for other E3-ubiquitin ligases [17,18].

### Hakai overexpression enhances invasiveness

**Figure 5:**
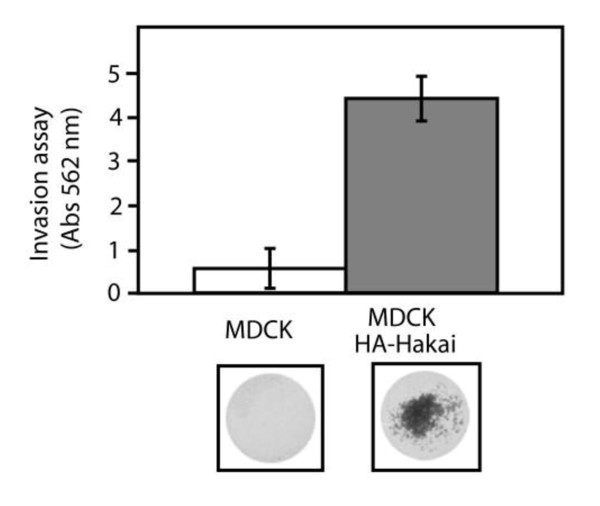
**Effect of Hakai overexpression on cell invasion**. Parental and Hakai-overexpressing MDCK cells were plated in an invasion chamber. After 24 h of incubation, invaded cells were stained with crystal violet and the absorbance of the stained cells was measured. Lower panels represent the staining of the migrating cells through the extracellular matrix. Data are represented as the means ± SD.

In closing, we examined whether the overexpression of Hakai also affect the invasiveness of cells. We used a polycarbonate membrane over which a thin layer of extracellular matrix (ECM) was applied to serve as an in vitro basement membrane. Since only invasive cells are capable of migrating through the ECM layer, we analyzed the ability of parental and Hakai-overexpressing MDCK cells to invade this *in vitro *matrix. Staining of the cells that passed through the membrane revealed that, whereas parental cells did not invade the matrix, Hakai-overexpressing cells readily passed through the membrane (Figure 5), indicating that overexpression of Hakai also enhances cell invasion. Taken all together, these observations suggest that Hakai may be involved in the regulation of invasion and cell-substratum adhesion. However, further investigations of Hakai in *in vitro *and *in vivo *model systems would lead us to validate its role during tumorogensis. Therefore, Hakai is implicated in several processes that are often occurring during epithelial-mesenchymal transition (EMT). In the last few years, it has been proposed that EMT mRNAs markers can be detected in circulating tumour cells (CTC) in the blood from cancer patients, helping to select new therapeutic targets with important clinical implications [19] Indeed, our group has recently proposed Plakophillin-3 mRNA, involved in epithelial cell-cell adhesions, as a biomarker for detection of CTCs in gastrointestinal cancer patients [20]. Further investigation is warranted to elucidate the molecular mechanism whereby Hakai influences these processes under physiological and pathological conditions, and to assess the potential usefulness of Hakai as therapeutic target for cancer.

## Conclusions

In summary, we present evidences that indicate that Hakai is implicated in the regulation of cell-substratum adhesions and in epithelial cell invasion. We have shown that the overexpression of Hakai in epithelial cells reduces Paxillin expression level and it disappears from focal adhesions and focal contacts. We demonstrated that this Paxillin reduction can not be recovered by using two important proteasome inhibitors, such as lactacystin and MG132, further supporting that Paxillin down-regulation is controlled by a proteasome-independent mechanism. Furthermore, we have shown that paxillin reduction may, at least partially, result from decreased expression level of E-cadherin. All these data suggest the implication of Hakai in controlling several important cellular processes that are crucial during tumour progression.

## Competing interests

The authors declare that they have no competing interests.

## Authors' contributions

AF, TRR and MCC contributed to experimental research. AF contributed to the design, the analysis of the results and writing the manuscript. All the authors have given the discussion, critical reading of the manuscript and final approval of the version to be published.

## Pre-publication history

The pre-publication history for this paper can be accessed here:

http://www.biomedcentral.com/1471-2407/11/474/prepub
